# Chit-Chat

**Published:** 1880-01

**Authors:** 


### Editorial Chit-Chat

SUBSCRIPTION DUE.—Send in your fifty cents for the coming year. Three, two, and one cent stamps received. Do not send stamps of a higher denomination.

MICROSCOPE FOR SALE.—We offer for sale an excellent microscope, as good as new, the stand all brass, Pike's make, with two eye pieces and two objectives, giving a power from 50 to 950 diameters, all for sixty dollars. It is an instrument which we have used in the finest of investigations, and will warrant it to show the fine lines in the *suerella gemma*. The instrument cannot be purchased of any dealer, second hand, for less then $150.

We have ordered a more costly instrument, for use with higher powers, and have no use for this one, hence the reason in offering it for sale.

AN INSCRIPTION.–As the newspapers throughout the country are just now busy suggesting inscriptions for our Adam monument, the BISTOURY presents one, to-wit:

ADAM,<br>

The Great Father of the Human Race,<br>

Evolved from Protoplasm<br>

In the Beginning of the World,<br>

And died at the ripe age of<br>

930 Years !<br>

This Monument is Erected by His Descendents<br>

Residing in Elmira,<br>

In the year 1880.

THE NEW COVER.—Of course, our friends have not faled to admire our new cover. It was designed by Mr. Fred. E. Murray, the same artist who so successfully illustrated our book, BODINES. The knife which rests upon the word BISTOURY is itself a bistoury, illustrating the very useful instrument after which our magazine is named. The entire design is artistic and beautiful. We know of none ornamenting a magazine that approaches it as a work of art.

The table of contents will be found on the inside of the front cover. Otherwise, the arrangement of the journal is the same as before.

Let us remind our readers that very many of them are far in arrears with their payments to the BISTOURY. Do not think we have forgotten it. A day of reckoning and settlement will come.

To our thoughtful and always punctual subscribers, the enclosed blank will be sufficient notice that subscription for another year is due. If you have not fifty cents, enclose a dollar—the sum is small enough—and we will credit you with two years' subscription instead of one. If you send postage stamps (which are as good as cash), do not send those of a higher denomination than three cents.

TELEGRAPH BOYS.—They are great conveniences, are those messenger and telegraph boys. We would dislike exceedingly to be deprived of their services. We have just one fault to find with them. What need is there, when you give them a message to carry to the office, to stand before you and read every word of it aloud, that all in the room may hear ? Will the message go any the faster or the more correctly for the boy's proclamation of it ?

Now and then one sends a dispatch, the contents of which he does not want known, and yet that little booby will stand there and solemnly read every blessed word of it to the attentive listeners, then toddle off as though he had accomplished a wonderful feat. We protest against the usage.

THE PENNY NUISANCE AT SCHOOL.—One of the most provoking of annoyances is to have one of your numerous progeny break in upon you in your private study, almost daily, with :

"Pa, please give me a penny for paper !"

If it isn't for paper, then it's for pens, ink, pencil ; or ten or fifteen cents for a copy book, drawing book, or the like. It is not the money one cares for, but the nuisance of being interrupted so often for those petty claims. What a convenience it would be to have our schools absolutely free. Not only free tuition, but free books, paper, pencils, ink, &c. It would not only do away with a great annoyance to the parent, but to the teacher also, who frequently is greatly inconvenienced by not having these articles supplied them when they are greatly needed. Some parents are negligent or forgetful of the wants of the children at school, others are really unable to supply what is absolutely needed for their children's advancement. It were better, therefore, for all concerned, that we should tax ourselves for all the paraphernalia used in our schools, and have them supplied to the scholars, free, as needed. A deal of annoyance, to all parties concerned, would thus be avoided.

THAT SALAD CONTEST.—It occurred upon East Hill, at an Institution famous as a resort for invalids, and at intervals, for those not so sick. A challenge was made by one of the physicians that she could name a certain eminent divine who would make a better potato salad than any other mortal. The other doctor —he of the spectacles and spicy pen, declared that he could name a chap, in the city, that could "double discount" the clergyman (whatever that is), at that game. So the hosts were gathered, doctors, lawyers, artists, travelers, and other distinguished and witty people, of both sexes. They arrayed themselves about the long table in the dining-room of the institution of health and jollity, when the trial commenced. The two contestants were seated at the middle of the table, opposite each other, and, while they were busy with the mysterious combination of oil and egg, the merry lookers' on fired their unmerciful witticisms at them and each other. The doctor with unpronounceable name, was in his element—or the element in him, blamed if we could tell which, for he was irrepressible in "keeping the table in a roar." The ladies showered us with their brilliant puns, and uttered their witty little speeches, while the traveler made pointed and pithy comments as he found opportunity, between. At last, the two salads were completed, and an umpire selected to decide upon their respective merits. We wouldn't have been in that judge's place for one of Spencer's 75th objectives, or a Zentmayer stage, (and they are two of the most desirable articles we can think of at this present moment), for, those two ladies who fed the judge, on either side, placing his eyes, ears, and nostrils to an entirely new use—not laid down in works of physiology of which we have any knowledge—gave him more potato salad than he ever dreamed of assimilating even in his wildest fancy of gastronomic exploits. We would be willing to wager the two articles above coveted that he was homesick if not stomach-sick after the feeding process had been completed. But, to his everlasting credit, be it said, he made a decision—decided in favor of the *other* fellow, though for why, no one could find out, and he was too full to give any intelligible explanation. The only moral to be drawn from all this is, (we have been wondering where we *would* land) if you would have fun,—real, sparkling, intellectual fun, invite a party of ladies and gentlemen to a potato-salad contest. It satisfies the stomach and intellect so thoroughly that nothing is left worth the coveting.

---

A FREE LOVE MARRIAGE.—Some time ago —no matter when, a man and woman appeared before a certain clergyman of this city—you might easily guess who—and demanded a marriage ceremony. The minister, seeing they were strangers, informed them that he could not marry them without being satisfied that they were legally entitled to be married. They could give no references in our city, said they came from Kentucky and knew no one nearer than Louisville. They were of suitable age for marriage, so the minister proposed questioning them, apart from each other, to see whether they would tell the same story. To this they assented. The man was taken aside and gave satisfactory responses to questions asked him, and accounted for his coming so far for a marriage ceremony. The woman told the same story, so the minister concluded to marry them.

"I want you to distinctly understand, sir," said the woman to the clergymen, before they arose to enter the parlors where the marriageable man was in waiting, "I want you to know, indeed, that I take no stock in ministers or religious observances. I simply come to you to make a contract to live with this man."

"You could have had it accomplished cheaper and so saved me a great annoyance, by having gone to a Justice of the Peace," replied the minister.

"O, we're perfectly able to pay for what you do for us, but I want you to know my sentiments—I'm lecturing on what you ministers call "free love," but what we women of brains call the right to control our own bodies," she said, giving her long curls a flop to the opposite shoulder.

"You want to contract to live with this man, then, until you become tired of each other, then to separate and try some other affinity—is that your purpose? queried the minister.

She assured him that was about her idea of the marriage relation. They now reached the parlor, when the minister anxious to get the job off his hands, commanded them to stand up. Addressing the man he enquired:

"Do you take this woman to be your lawful wedded wife?"

"I do," was the quick response.

"My dear sir," continued the minister, "I tell you frankly, *you had better go to hell* than marry this woman; however madam," addressing the woman,

"Do you take this man for your husband ?

"I do."

"Well, all I can say is, you are man and wife, and you will both regret it as long as you live —go !"

The man offered the minister a ten dollar note for the "ceremony," which he promptly refused, saying :

"No, sir, I don't want your money, it is too nasty a job to receive pay for ; go, and may the good Lord have mercy upon both of you!"— And they went.

---

GOT THE BEST OF HIM.—We had just devoted a straight hour to convincing a book agent that we did not need, neither would we purchase his book ; had dismissed an agent with some new-fangled concern the uses of which it took another hour to explain. We did not begrudge the last mentioned hour, because it gave us an inspiration—a new light dawned upon us, which we were prepared to put into practice when the third fellow entered our door. Three agents, in one morning, to a busy man, was exasperating. We resolved to devote the remainder of the day to this chap, and give him a dose of his own physic, just to see how it would set on his stomach.

To his salutation, "are you busy, sir ?" we smilingly replied, "O, no, not at all, sir ; got the entire day before me—what can I do for you ?"

He drew nearer, opened an immense valise which had been carried in by a colored boy (the nobby ones all travel with a colored man now), spread it out, and proceeded to enlighten us upon the great utility of a certain instrument which all physicians should possess.

"Almost similar to one of mine," we replied, walking toward our instrument case, and lifting its lid ;—"here is an instrument invented by the great surgeon Dr. Gross," we continued, "it is wonderfully well adapted to its purpose; see how neatly that cylinder moves within its fellow—you cannot see where they join," holding it up to the light and expatiating upon its uses for a straight hour and a quarter, by the watch.

During our lecture upon the instrument, two little girls glided into the room, and, seeing the agent's bright instruments spread out bewitchingly upon the floor, proceeded to convert them into "play-things," while we held our victim to his work. He was about to pick up his articles when we called his attention to another curious instrument, a lithotrite, and commented upon it for another three quarters of an hour, until the sweat upon the agent's face stood out in great beads, betokening his delight with the subject in hand. (Just then, one of the little girls walked out into the street, dragging one of the agent's beautifully silvered instruments by a string, thus improvising a cart.) Seeing that our victim was growing pale at the mere recital of the uses of a lithotrite, we quickly took up an aspirator needle ; showed him how we could thrust it into an abcess of the liver and draw out the matter, push it into the lungs and wiggle it around there in search of pus, water, or anything else he was in need of. Showed him, on his own body, the spot where we thrust it between the ribs to get at the heart, in order to remove any superfluous water that is there suspected to be deposited. As we placed our finger upon the fifth intercostal space, and inclined the needle in the direction it should take, making a feint as though about to puncture his own heart, he grew deathly pale, his eyes closed, he gave a sigh, and sank helplessly into a chair, while his arms fell lifelessly by his side. We saw his extreme interest in our theme, and the advantage we had gained, so we held him in his chair with one hand and secured a trephine with the other. This unique little instrument we held before his deathly face, and showed him how we bored into a man's skull with it ; described how the brain sometimes protruded through the wound to the size of a hen's egg ; then picking up a long and formidable knife, exhibited to his glassy eyes how we shaved this gob of brain matter off, giving a great swoop of the the knife over his head in illustration of the process. Then we shouted in his ear (for he seemed to be unconscious now), "then, you see, if the man lives, we——" but it was no use to pursue this interesting subject further. The poor fellow had fainted away entirely at the bare imagery of our last remark. That fixed him. He slid from the chair to the floor, where we left him to the tender care of the assistant, who dashed water into his face to restore him to consciousness. We left directions for him not to be in a hurry to leave, that we would soon return and tell him some more real pretty little stories. But, strange to say, he didn't wait, and when our little girl returned with her go-cart, we felt an inward satisfaction of having got the best of at least one tantalizing agent.

